# Indicators of mortality risk in ageing horses

**DOI:** 10.1007/s11357-025-01738-y

**Published:** 2025-06-25

**Authors:** Z. Kelemen, C. Vogl, L. Torres Borda, U. Auer, F. Jenner

**Affiliations:** 1 Equine Surgery Unit, Centre for Equine Health and Research, Department for Small Animals and Horses, University of Veterinary Medicine Vienna, Vienna, Austria; 2 Department for Biological Sciences and Pathobiology, University of Veterinary Medicine Vienna, Vienna, Austria; 3 Anaesthesia Unit, Centre for Small Animal Health and Research, Department for Small Animals and Horses, University of Veterinary Medicine Vienna, Vienna, Austria

**Keywords:** Mortality, Equine, Quality of life, Weight loss, Appetite, Pain, Depression

## Abstract

Clinical care for patients with limited life expectancy often requires adjustments, prioritizing immediate benefits over long-term outcomes, as the relevance of future complications diminishes. This study identifies indicators of mortality risk in horses with chronic orthopaedic conditions to enhance individualized care and welfare. Over 3 years, 123 chronically lame horses and 6 healthy control horses at an animal sanctuary underwent regular (every 3 months) comprehensive health assessments and activity monitoring using wearable sensors. Data collected included body condition scores, musculoskeletal pain scores, lameness evaluations, and time budgets for eating, resting, and activity. Of the 123 chronically lame horses, 31 horses died (*n* = 31/123, 25.2%), with 10 succumbing to acute decompensation of their chronic condition (DAC, *n* = 10/123, 8.1%), while 21 were euthanized due to intractable pain or progressively deteriorating health and function (DCC, *n* = 21/123, 17.1%). Statistical modelling using death as outcome measure revealed body condition, pain scores, and time budget data to be strongly associated with equine mortality. Notably, low body condition score and reduced eating time predicted mortality in DAC horses, aligning with human studies linking weight loss to frailty and increased mortality risk. Additionally, depression-like behaviours were prevalent in DAC horses, mirroring the link between depression and mortality in humans. While pain scores were elevated in all deceased horses, weight loss was specific to DAC, suggesting multifactorial influences beyond pain. These findings provide a foundation for developing equine-specific tools to predict outcomes and guide clinical and end-of-life decisions, enabling individualized treatment to enhance the welfare and quality of life for aging horses. These insights may also offer valuable information for human medicine, particularly for at-risk groups such as individuals with cognitive impairments who may struggle to communicate their symptoms.

## Background

Increased life expectancy stands as both a triumph and a challenge of modern medicine. Advances in medical care have extended lifespans, leading to a growing geriatric population in both humans and horses [1–8], currently accounting for 21.3% of the human and 11.4–34% of the equine population [6, 7, 9–11]. However, increased lifespans are not always accompanied by corresponding gains in health spans. Aging is frequently associated with an accumulation of chronic conditions that limit physical function, contribute to persistent pain, and drive frailty, morbidity, and escalating healthcare needs [1–8].

Clinical care and decision-making may require adjustments for patients with a short residual life expectancy, for whom the immediate side effects of preventive treatments may outweigh their long-term benefits, and potential future complications of palliative treatments become less relevant [12–16]. This shift supports the use of more aggressive pain management than would typically be considered appropriate for patients likely to live long enough to experience such complications. Consequently, treatment plans should consider the remaining life expectancy to optimize the quality of life. However, many elderly individuals retain sufficient physical and functional resilience to benefit from medical procedures like younger patients [12–16], emphasizing the importance of tailoring clinical decisions to each individual by carefully balancing the risks of adverse outcomes against the expected benefits. Yet, accurately estimating life expectancy or mortality risk remains challenging in clinical practice [17–20]. As a result, treatment decisions are often based on chronological age rather than a comprehensive assessment of a patient’s fitness and biological age [3, 4, 12–16, 21–26]. The significant heterogeneity in ageing rate underscores the need for prediction models that assess short- and medium-term mortality risks using objective indicators to support clinical decision-making and optimize individualized treatment.

Functional ability measures, such as Timed Up-and-Go (TUG) and gait speed, are strong predictors of mortality in elderly humans [12–16] but their clinical integration faces resistance due to time and space constraints, as well as clinicians’ unfamiliarity with these methods. Furthermore, single time-point assessments are often inadequate, as physical activity and functional measures are influenced by performance biases, circadian rhythm, and seasonal fluctuations and typically decline gradually over extended periods. This slow decline necessitates repeated longitudinal observations, which are further complicated by logistical challenges. Although continuous monitoring via wearable sensor technology could provide an efficient and objective reflection of daily activities, privacy concerns remain a significant barrier to its implementation in human patients [27–32].

In contrast, equine functional assessments and biological age markers remain to be established to provide critical insight into the aging process and mortality risks in horses, thereby enhancing equine welfare. Identifying these indicators would not only allow veterinarians to implement timely and individualized interventions, potentially improving quality of life for aging horses, but it would also facilitate preventive care strategies and optimized palliative management for elderly horses. Additionally, these assessments may help distinguish between age-related functional decline and other disease processes, enabling more accurate diagnoses and treatment plans.

In horses, continuous monitoring of activity time budgets is feasible and widely used in welfare assessments [31–34], providing a valuable opportunity to go beyond the parameters established for human patients and identify objective markers of impending mortality.

Therefore, this study uses longitudinal data from regular health assessments and multi-day activity monitoring to identify key health parameters and activity patterns that may serve as reliable predictors of mortality risk in horses. The goal is to develop functional assessment tools for health trajectories in aging horses and support personalized preventive care strategies for equine patients.

## Materials and methods

### Horses, housing, and management conditions

This prospective, observational cohort study followed a population of 129 mixed-breed horses housed at an animal sanctuary over 3 years. The primary study cohort consisted of 123 horses (58 geldings, 65 mares), aged 4–34 years (mean age, 21.3 years; s.d., 5.7 years), with chronic (> 6 months) orthopaedic conditions defined as a lameness score > 1 on the American Association of Equine Practitioners (AAEP) scale. For comparison, a control group of six younger horses (2 geldings, 4 mares), aged 4–16 years (mean age, 10 years; s.d., 4.7 years), without chronic health issues was included.

Horses had daily turnout in either paddocks or fields, depending on seasonal and weather conditions. Water was provided ad libitum, and all horses were fed a hay- or grass-based diet. In the stables, which were bedded with shavings, hay was provided twice daily, in the morning and afternoon. During paddock turnout, hay was available freely via 12-slot feeders. While in the fields, horses grazed without additional hay supplementation.

### Health parameters and time budget analysis

Every 3 months, each horse underwent a comprehensive physical and orthopaedic examination, body condition scoring (BCS[35]), and a standardized, validated pain assessment (MPS[36]). These examinations were all performed by the same veterinarian to ensure consistency. Additionally, time budgets for eating, resting, and general activity were recorded continuously over five-day intervals using Hoofstep® wearable sensors [31, 33]. These devices, equipped with GPS, accelerometers, gyroscopes, and radio transmitters, were securely fitted on each horse’s forehead using a specially designed flexible softshell head collar. Data from the accelerometer, gyroscope, and an integrated AI algorithm classified the horse’s behaviour into one of four categories: (1) ‘eating’ (time spent chewing in any position or in combination with other behaviours), (2) ‘resting’ (without distinction between lying and standing), (3) ‘active’ (slow locomotion, such as walking), and (4) ‘highly active’ (fast movement, such as trotting or cantering, as well as potential stress behaviours like headshaking). These data are presented as the proportion of time spent in each of the four behaviour categories. Since the sum of these proportions is always one, negative correlations between states occur inherently. This effect is particularly pronounced for the two most common behaviours: eating and resting. Additionally, ‘activity count’ quantified transitions between behavioural categories over time.

### Death and corresponding grouping for analysis

Over the study period, 31 horses with chronic orthopaedic disease either died or were euthanized due to progressive health deterioration. Each of these horses underwent examinations and time-budget analysis within 100 days before death. For analysis, the chronically lame horses were divided into three groups: (1) horses that died or were euthanized periagonally due to an acute deterioration in their chronic condition (DAC, *n* = 10), (2) horses euthanized after a carefully deliberated decision due to a prolonged inexorable decline in health and function or intractable pain refractory to treatment (DCC, *n* = 21), and (3) horses that survived until the conclusion of the study (NC, *n* = 92). These groups were compared to each other and to the control group of young, healthy horses (Y).

### Statistical analysis

Statistical analyses were conducted using R (version 4.2.2) and GraphPad Prism (version 9.5.1), with the significance level set to *α* = 0.05. Various descriptive statistics and graphics were used to summarize the data. An ANOVA with Tukey’s multiple comparisons test was used to compare differences between groups for health parameters and time budgets, based on the final examination results of the horses prior to euthanasia (for DAC and DCC) or at the end of the study (for NC and Y) to detect differences in parameter means between groups. Additionally, for each horse with more than one examination, the difference between the last and second-to-last examination results was calculated, along with the ratio of this difference to the average of all previous measurements.

To investigate whether the fate of ageing horses can be predicted from their examination and time budget data, multinomial logistic regression was used to assess associations between clinical and behavioral variables and categorical mortality outcomes (N, DAC, DCC). This method models the probability of each outcome as a function of explanatory (regression) variables, allowing identification of the factors that significantly influence the likelihood of each specific mortality outcome. The following explanatory variables were included in the model: the body conditions score (BCS), the musculoskeletal pain score (MPS) summarizing its subitems, lameness in walk and the trot, the proportion of time spent eating, resting or active, the activity count (= number of behaviour changes), and the number of chronic diseases. We note that the influence of the variables representing the proportion of the time budget must be interpreted with caution due to their inherent correlations. As sex and differences in age between the horses may confound the analysis, both variables were included as controls. In addition, binomial logistic regressions were conducted contrasting (1) DAC versus NC horses and (2) deceased (DAC and DCC) versus NC horses, as dichotomous outcome variables allow analyses of sensitivity, specificity, and receiver operating characteristic (ROC). ROC analysis evaluates the predictive performance of a model by plotting the true positive rate (sensitivity) against the false positive rate (1—specificity) across a range of threshold values. The area under the ROC curve (AUC), also referred to as the concordance statistic (c-statistic), provides a summary measure of the model’s discriminatory ability. It represents the probability that a randomly selected case will have a higher predicted risk than a randomly selected control. AUC values range from 0.5 (no discrimination) to 1.0 (perfect discrimination) and are typically interpreted as indicating low (0.5 < c ≤ 0.7), moderate (0.7 < c ≤ 0.9), and high (c > 0.9) test accuracy [37–39]. Our choice of variables is a compromise between interpretability (e.g., inclusion of age and sex as control variables) and the AIC values in the three analyses.

## Results

### Horses, examination parameters, and time budgets

Horses were examined between 1 and 11 times (mean, 6.7; s.d., 3.3) with examination intervals ranging from a minimum of 0 days to a maximum of 89 days (mean, 31.87 days; s.d., 25.16).

Horses in the primary cohort, for which chronic lameness was an inclusion criterion (DAC, DCC, NC), predominantly suffered from osteoarthritis (*n* = 72), tendinopathy (*n* = 27), and laminitis (*n* = 22). For ten horses, the cause of chronic lameness was unknown. Additionally, some horses presented with chronic respiratory (*n* = 8), cardiac (*n* = 9), metabolic (*n* = 19), ophthalmologic (*n* = 9), and dermatologic (*n* = 23) conditions. Horses in the DAC group had an average of 2.2 chronic conditions (± 1.135), which was significantly higher than the NC group (1.34 ± 0.579, *p* = 0.0003) and marginally higher than the DCC group (1.67 ± 0.658, *p* = 0.0875). The difference between the DCC and NC groups was not significant (*p* = 0.0952). Horses in the DAC, DCC, and NC groups were significantly older than those in the Y group (*p* < 0.0001); however, there was no significant age difference among the three groups with chronic conditions (Table 1).

**Table 1:** Descriptive statistics of the health parameters and activity time budgets of each horse last examination, detailed by group into horses with chronic diseases that died acutely (DAC), were euthanized after failure to respond to treatment (DCC), or are still alive (NC) and a control group of horses without chronic health problems (Y)

	***DAC***		***DCC***		***NC***		***Y***	
	*(n* = *10)*		*(n* = *21)*		*(n* = *92)*		*(n* = *6)*	
	*Mean*	*s.d*	*Mean*	*s.d*	*Mean*	*s.d*	*Mean*	*s.d*
Age	*23.3*	*6.0*	*23.2*	*6.0*	*20.7*	*5.5*	*10.0*	*4.7*
Chronic conditions	*2.2*	*1.1*	*1.7*	*0.7*	*1.3*	*0.6*	*0*	*0*
Change in health	* − 0.2*	*0.6*	* − 0.8*	*0.5*	* − 0.1*	*0.4*	*0*	*0*
Musculoskeletal pain score	*9.3*	*3.5*	*11.1*	*3.3*	*6.2*	*3.0*	*1.0*	*0.9*
Body condition score	*5.2*	*1.8*	*5.1*	*1.5*	*6.8*	*0.7*	*7.0*	*0.0*
Lameness in walk	*2.5*	*1.4*	*3.1*	*1.1*	*1.6*	*0.7*	*1.0*	*0.9*
Lameness in trot	*3.1*	*1.8*	*4.5*	*1.7*	*2.0*	*1.1*	*1.2*	*0.8*
Eating (%)	*31.9*	*7.3*	*40.9*	*10.1*	*39.4*	*7.1*	*37.0*	*4.3*
Resting (%)	*48.1*	*8.1*	*41.7*	*9.0*	*42.7*	*7.8*	*39.7*	*8.7*
Active (%)	*10.5*	*4.6*	*8.4*	*3.1*	*8.7*	*3.2*	*10.8*	*1.9*
Highly active (%)	*9.5*	*5.5*	*9.1*	*4.6*	*9.2*	*3.2*	*12.5*	*8.9*
Activity count	*490.1*	*475.5*	*430.2*	*204.0*	*534.9*	*338.7*	*583.5*	*207.6*
Recumbency (min)	*37.5*	*40.8*	*104.4*	*97.3*	*47.0*	*41.0*	*69.8*	*44.6*

### Health parameter and time budget analysis

**Fig. 1:**
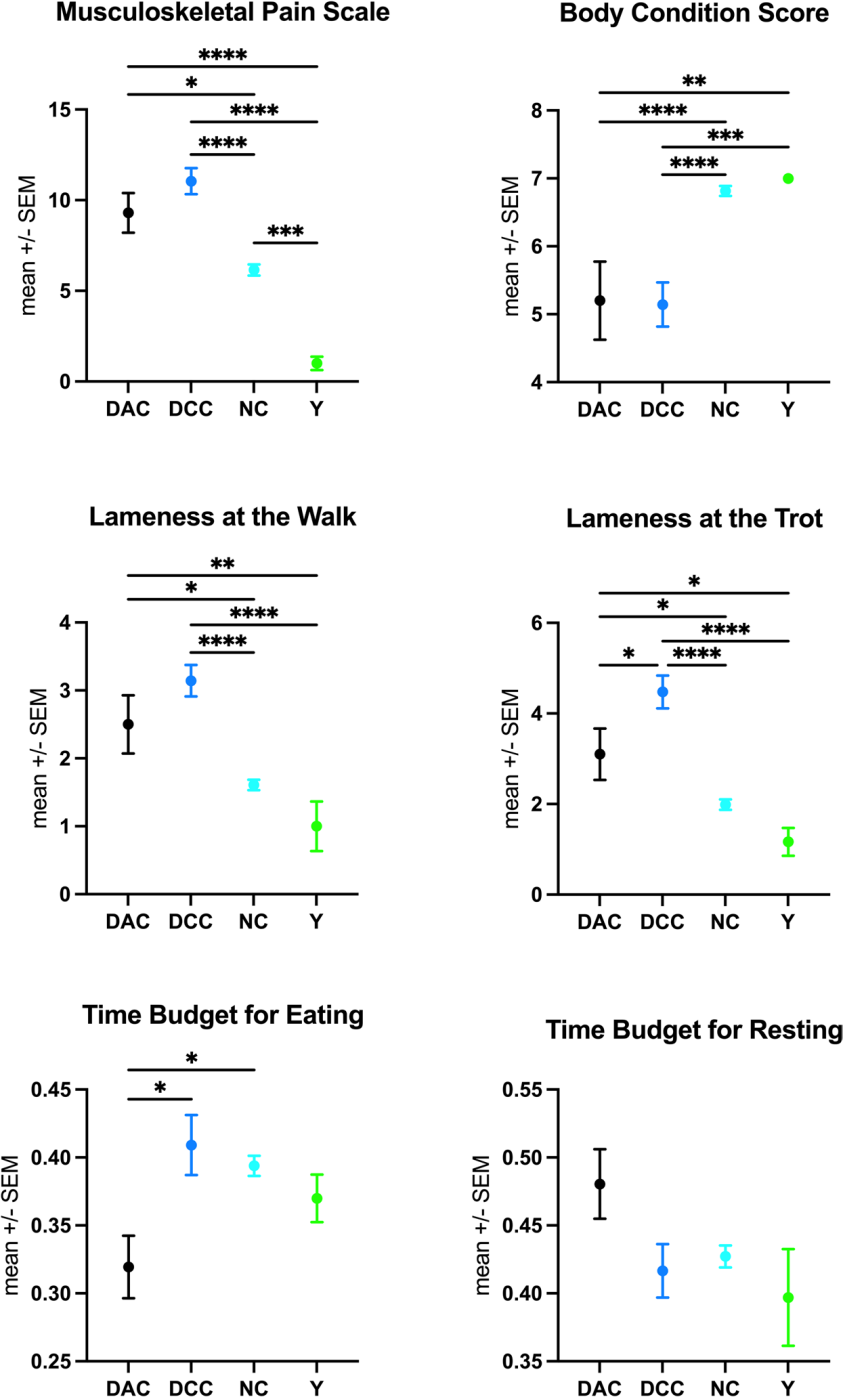
Comparison of health parameters and activity time budgets across different groups: the healthy control group (Y) and horses with chronic orthopaedic conditions that (1) died or were euthanized due to acute deterioration (DAC), (2) were euthanized after careful consideration due to irreversible decline in function or intractable pain unresponsive to treatment (DCC), and (3) survived to the end of the study (NC). Measurements were compared using ANOVA with Tukey’s multiple comparison test (*p < 0.05, **p < 0.01, ***p < 0.001, ****p < 0.0001). Low body condition scores and reduced eating time budgets emerged as significant indicators of equine mortality in this study. DAC horses spent significantly less time eating than DCC (*p* = 0.0136) and NC horses (*p* = 0.02). In addition, horses in the DAC and DCC groups showed significantly higher pain (MPS) and lameness scores in both walk and trot than surviving horses (NC). While no significant differences in pain scores were observed between the two deceased groups (DAC mean, 9.3; DCC mean, 11.05), DCC horses had the highest lameness scores at the trot (compared to DAC: *p* = 0.0263, NC: *p* < 0.0001, Y: *p* < 0.000) and the greatest deterioration in overall health status (compared to DAC: *p* = 0.0071, NC: *p* < 0.0001, Y: *p* = 0.0018)

Horses in the DAC and DCC groups showed significantly higher pain (MPS) and lower body condition (BCS) scores than horses in the NC and Y groups (*p* < 0.0001, Fig. 1, Table 1). Additionally, they had significantly higher lameness scores in both walk and trot compared to the NC and Y groups (*p* < 0.0001). Notably, there were only a few significant differences between DAC and DCC. Specifically, DAC horses spent significantly less time eating than DCC (*p* = 0.0136) and NC horses (*p* = 0.02), while DCC horses showed the highest lameness scores at the trot, being significantly lamer than NC (*p* < 0.0001), Y (*p* < 0.0001), and DAC horses (*p* = 0.0263). DCC horses also had the greatest deterioration in overall health status, which was significantly worse than DAC (*p* = 0.0071), NC (*p* < 0.0001), and Y (*p* = 0.0018, Fig. 1, Table 1), likely contributing to the decision for euthanasia.

While the overall pain scores did not differ significantly between DAC horses (mean, 9.3; s.d., 3.5) and DCC horses (mean, 11.05; s.d., 3.3; *p* = 0.4333), specific subitems of the scale revealed distinct patterns of discomfort and behavioural change. DCC horses exhibited significantly higher scores for ‘head-neck-withers alignment’ than any other group (DAC: *p* = 0.0043; NC: *p* < 0.0001; Y: *p* = 0.0008), indicating pronounced musculoskeletal discomfort. Conversely, DAC horses had significantly higher scores for ‘position in their enclosure’ (DCC: *p* = 0.0071; NC: *p* = 0.0022; Y: *p* < 0.0001), suggestive of apathy and disengagement from their surroundings. They also scored significantly higher for ‘demeanour’ (DCC: *p* = 0.0273; NC: *p* < 0.0001; Y: *p* < 0.0002, Suppl. Figure 1), reflecting depression and withdrawal.

In contrast, the NC and Y groups differed only in pain scores, with NC horses showing significantly higher pain levels than Y horses (*p* = 0.0005), particularly in the musculoskeletal pain scale’s subitems posture (*p* = 0.0002), lameness (*p* = 0.029), and enclosure position (*p* = 0.019).

**Fig. 2:**
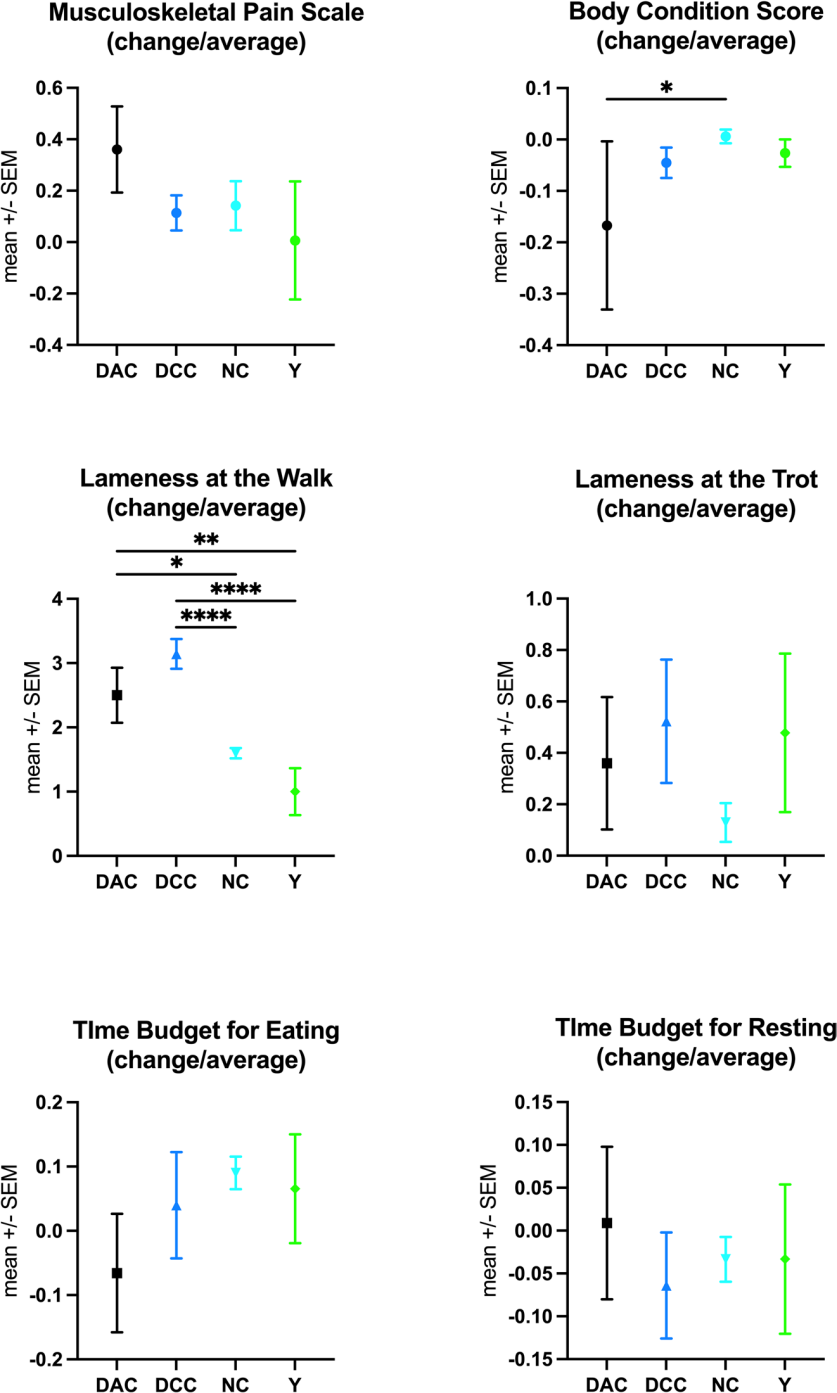
Assessment of the trajectory of health parameters and activity time budgets. For each horse, the ratio of the difference between the last and second-to-last measurements to the average of all previous measurements was calculated for each parameter. This ratio was compared across groups using ANOVA with Tukey’s multiple comparison test (*p < 0.05, **p < 0.01, ***p < 0.001, ****p < 0.0001). The groups compared include the healthy control group (Y) and horses with chronic orthopaedic conditions that (1) died or were euthanized following an acute deterioration of their condition (DAC), (2) were euthanized after a careful decision due to inexorable decline in function or intractable pain unresponsive to treatment (DCC), and (3) survived to the end of the study (NC). DAC horses showed a significant decrease in BCS compared to NC (*p* = 0.047) and an increase in lameness in walk compared to NC (*p* = 0.0124) and Y (*p* = 0.0062). DCC horses, however, exhibited the most pronounced increase in lameness in walk compared to NC and Y (*p* < 0.0001) and showed a tendency for increased lameness compared to DAC (*p* = 0.226)

Significant changes in health parameters and time budgets between the second-to-last and last measurements, relative to previous averages, were observed only for BCS and lameness in walk (Fig. 2). DAC horses showed a significant decrease in BCS compared to NC (*p* = 0.047) and an increase in lameness in walk compared to NC (*p* = 0.0124) and Y (*p* = 0.0062). DCC horses, however, exhibited the most pronounced increase in lameness in walk compared to NC and Y (*p* < 0.0001) and showed a tendency for increased lameness compared to DAC (*p* = 0.226, Fig. 2).

### Indicators of mortality risk

Multinomial logistic regression analysis predicting the fates of horses (DAC, DCC, and NC) showed only few variables with significant influences (using the criterion of zero not being overlapped by mean plus/minus two times the standard error, SE) between the chronically lame horses dying naturally (DAC) and those being euthanized (DCC). In particular, DCC horses spent less of their time active and resting than DAC horses (Table 2), while eating showed no significant difference. As eating, active, and resting make up nearly all the time budget of the horses (horses are rarely very active), the DCC horses must spend more time eating than the DAC horses, which is only apparent when also considering the higher intercept of the DCC group (Table 2). Many more parameters significantly influence the distinction between DAC and NC: Indeed, of the 12 variables, only sex, age, lameness in the trot, and activity count do not, and of these, the former two are control variables and the other two variables are likely correlated with variables that show a significant influence (Table 2).

**Table 2:** Multinomial logistic regression: estimates and standard errors of predictors of differences between horses with chronic orthopaedic disease that died (DAC) and survivors (NC) as well as horses with chronic orthopaedic disease that died (DAC) and horses that were euthanized due to a prolonged inexorable decline in health and function or intractable pain irresponsive to treatment (DCC); as a proxy to statistical significance, boldface indicates that the estimate plus or minus twice the standard error does not overlap zero

Predictor variable	Estimate DCC	SE DCC	Estimate NC	SE NC
Intercept	13.81	2.9	− 46.9	3.144
Sex	− 1.406	1.198	− 1.877	1.194
Age	0.1049	0.1123	0.3104	0.1139
BCS	− 0.1536	0.3679	**1.773**	**0.4226**
Musculoskeletal pain score	0.0176	0.204	− **0.4267**	**0.1909**
Lameness walk	− 0.0824	0.8661	− **2.1065**	**1.000**
Lameness trot	1.001	0.5848	− 0.1251	0.5487
Activity count	− 0.0028	0.0017	0.0011	0.0016
Time budget active	− **16.3**	**0.4256**	**66.72**	**0.2684**
Time budget resting	− **25.19**	**1.8023**	**43.79**	**2.499**
Time budget eating	− 3.313	2.627	**52.56**	**2.638**
Chron. diseases (*n*)	− 1.234	0.7077	− **2.617**	**0.659**

**Fig. 3:**
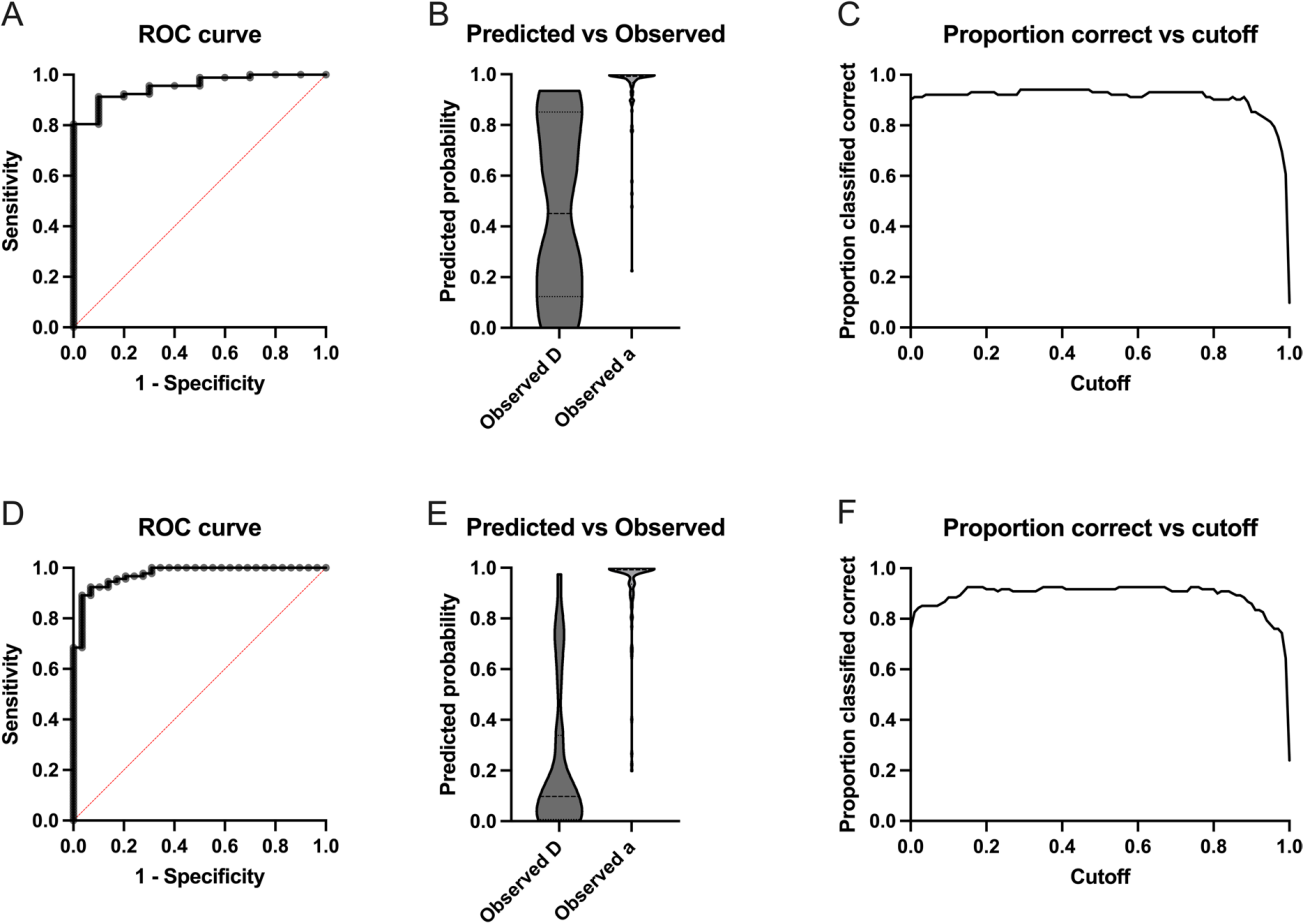
Binomial logistic regression analysis predicting mortality (death) based on age, sex, body condition score, discomfort score, lameness in walk and in trot, number of chronic health problems, and activity time budgets as explanatory variables. Top row (**A**, **B**, **C**): comparison of chronically lame horses that died or were euthanized periagonally (DAC) versus surviving horses (NC). Bottom row (**D**, **E**, **F**): all deceased (DAC and DCC) versus surviving horses. **A**/**D** ROC curve: The receiver operating characteristic (ROC) curve shows the model’s ability to discriminate between deceased and surviving horses. ROC curves plot the true positive rate (sensitivity) against the false positive rate (1—specificity) across a range of threshold values. The area under the curve (AUC) provides a summary measure of the model’s discriminatory ability (**A** AUC = 0.953, **D** AUC = 0.974). AUC values can range from 0.5 (no discrimination) to 1.0 (perfect discrimination) and are typically interpreted as indicating low (0.5 < c ≤ 0.7), moderate (0.7 < c ≤ 0.9), and high (c > 0.9) test accuracy. **B**/**E** Predicted vs. observed mortality outcomes: These plots compare the predicted probabilities of mortality generated by the logistic regression model to the actual observed mortality outcomes (D…dead, a…alive) for each horse, highlighting the model’s predictive accuracy. Ideally, a model with high predictive accuracy will show a clear separation between cases that resulted in death and those that did not, with higher predicted probabilities clustering around deceased cases. This visualization helps assess model calibration and the strength of association between the predicted risks and actual outcomes. **C**/**F** Proportion correct vs. cutoff: These graphs illustrate how the model’s overall classification accuracy varies depending on the selected probability cutoff value (threshold) for predicting mortality. At each threshold, the model classifies horses as likely to die or survive and calculates the proportion of correct classifications. The curve typically helps identify an optimal cutoff value that balances sensitivity (true positive rate) and specificity (true negative rate), thereby maximizing the model’s discriminative power

Reducing the data set to two groups allows for the application of a binomial logistic regression, for which further read-outs are possible. To assess the predictive accuracy of the various demographic, health, and behavioural parameters for mortality in chronically lame horses, we initially conducted a binomial logistic regression to differentiate between the DAC and NC groups. The DCC group was excluded from this analysis to avoid potential confounding, as euthanasia decisions in this group might have been influenced by the same data used as variables in the model. Analysis revealed the body condition score and the time budget for eating to significantly affect natural death (Table 3). In combination, the predictors resulted in a negative predictive value (true negatives among all negative predictions) of 71.43% and a positive predictive value (true positives among all positive predictions) of 94.74 (area under the ROC curve: AUC = 0.953, Fig. 3).

**Table 3:** Binomial logistic regression: estimates, standard errors (SE), confidence intervals (95% CI), and *p*-values of predictors of differences comparing horses with chronic orthopaedic disease that died (DAC) with survivors (NC)

Predictor variable	Estimate	SE	95% CI	*p*
Intercept	− 29.96	16.17	5.952e-031 to 0.3022	0.0638
Sex	0.05118	1.09	0.1192 to 10.47	0.9625
Age	0.2262	0.15	0.9647 to 1.792	0.1280
BCS	**1.298**	**0.55**	**1.412 to 13.57**	**0.0192**
Musculoskeletal pain score	− 0.3399	0.244	0.4055 to 1.104	0.1632
Lameness walk	− 0.9895	1.003	0.02916 to 1.930	0.3239
Lameness trot	− 0.02837	0.542	0.3325 to 3.339	0.9583
Activity count	1.530e-005	0.001	0.9974 to 1.003	0.9914
Time budget active	25.13	28.12	7.751e-012 to 1.252e + 039	0.3714
Time budget resting	23.60	17.23	0.0003233 to 4.380e + 027	0.1708
Time budget eating	**36.43**	**17.52**	**419.9 to 8.961e + 034**	**0.0376**
Chron. diseases (*n*)	− 1.324	0.824	0.04182 to 1.219	0.1078

A second binomial logistic regression analysis assessed the predictive accuracy of various demographic and health parameters and activity time budgets for mortality overall, i.e. combining horses that died naturally (DAC) and those euthanized by a veterinarian (DCC) into a single group, versus survivors (NC). The results identified body condition score, musculoskeletal pain score, lameness at the walk, the number of chronic health conditions, and the time budgets for eating, resting, and activity as significant predictors of mortality (Table 4). In combination, the predictors resulted in a negative predictive value of 85.19% and a positive predictive value of 93.62% (area under the ROC curve: AUC = 0.974, Fig. 3).

**Table 4:** Binomial logistic regression: Estimates, standard errors (SE), confidence intervals (95% CI), and *p*-values of predictors of differences comparing horses with chronic orthopaedic disease that died (DAC) or were euthanized due to a prolonged inexorable decline in health and function or intractable pain irresponsive to treatment (DCC) with survivors (NC)

Predictor variable	Estimate	SE	95% CI	*p*
Intercept	− 46.94	14.77	2.44e-36 to 3.814e-10	0.0015
Sex	1.045	0.945	0.502 to 23.14	0.2688
Age	0.207	0.114	1.001 to 1.594	0.0703
BCS	**1.672**	**0.450**	**2.501 to 15.73**	**0.0002**
Musculoskeletal pain score	− **0.388**	**0.186**	**0.444 to 0.946**	**0.0370**
Lameness walk	− **1.895**	**0.942**	**0.0143 to 0.698**	**0.0443**
Lameness trot	− 0.603	0.387	0.242 to 1.146	0.1190
Activity count	0.0023	0.002	1 to 1.006	0.1417
Time budget active	**67.72**	**26.56**	**1,431,865,862 to 3.239e + 055**	**0.0108**
Time budget resting	**46.34**	**14.62**	**1,476,841,168 to 1.103e + 035**	**0.0015**
Time budget eating	**46.14**	**15.31**	**544,864,561 to 9.886e + 035**	**0.0026**
Chron. diseases (*n*)	− **1.914**	**0.711**	**0.029 to 0.516**	**0.0071**

## Discussion

Low body condition scores and reduced eating time budgets emerged as significant indicators of equine mortality in this study. Analogously, weight loss and diminished appetite are well-established predictors of mortality risk in elderly humans, dogs, and cats without terminal illnesses [40–56]. Beyond serving as a prodromal marker of various life-limiting diseases, weight loss contributes to mortality both directly, through its immediate physiological impacts, and indirectly, by accelerating the development and progression of sarcopenia and frailty, conditions that independently increase mortality risk [25, 43, 54, 57–61].

Notably, in humans, weight loss is associated with an increased mortality risk regardless of baseline weight, even in overweight or obese individuals [40–42, 45–47, 53, 55, 56]. This phenomenon was also evident in this study where horses’ body condition scores ranged from ideal (BCS = 5) to overweight (BCS = 7), with only a few classified as thin. The equine body condition score is positively correlated with body weight, percentage of body fat, and plasma prostaglandin E2 (PGE2), making it a better indicator of adiposity and the associated increased systemic inflammation than body weight [35, 62]. While obesity in humans predisposes to sarcopenia, weight loss results in concurrent losses of not only body fat but also muscle and bone mass and may thus paradoxically exacerbate age-related sarcopenia and frailty [25, 43, 54, 57–61]. In older adults, where age-related body composition changes already lead to increased fat mass and reduced muscle tissue in both sexes [60, 63], such losses further impair physical function, deplete physiological reserves, and heighten vulnerability to stressors [25, 43, 54, 57–61]. Similarly, in geriatric horses, muscle loss can progress to the point where they are unable to rise from a recumbent position without assistance, a well-documented clinical problem. However, the etiopathogenesis of sarcopenia and frailty in horses has not yet been systematically studied, leaving a gap in our understanding of these conditions in the equine population.

The relationship between weight loss and mortality is more pronounced in men, where a > 10% weight loss is associated with a 289% increase in mortality risk compared to a 114% increase in women [40]. This disparity is attributed to body composition differences, as men typically have a higher muscle and bone mass, whereas women have a greater proportion of fat tissue [40]. In this study, no sex-specific differences were observed in horses, likely reflecting the similar testosterone levels in mares and geldings, both of which are significantly lower than those in stallions.

Poor appetite in the elderly, termed anorexia of aging, frequently results in inadequate food intake, leading to nutritional deficiencies and weight loss [40, 43, 50, 52, 54, 64–71]. Appetite regulation is a complex process orchestrated by the central nervous, endocrine, and sensory systems [40, 43, 50, 52, 54, 64–71]. In older adults, appetite reduction has been attributed to lower plasma levels of the orexigenic hormone ghrelin and elevated concentrations of insulin, leptin, cholecystokinin, and proinflammatory cytokines. Additional contributors include slower gastric emptying, altered taste and smell, dental issues, chronic pain, and psychological or social challenges [40, 43, 50, 52, 54, 64–71]. Ageing horses share many of these challenges, particularly chronic orthopaedic pain and susceptibility to dental problems. However, in this study, regular veterinary care, including routine dental examinations, ruled out oral health problems as a primary cause of weight loss.

While horses that died (DAC) or were euthanized (DCC) exhibited higher pain scores than surviving horses (NC), no significant differences in pain scores were observed between the two deceased groups (DAC mean, 9.3; DCC mean, 11.05). Interestingly, weight loss was observed exclusively in DAC, suggesting contributing factors beyond pain. Further studies examining associations between appetite-regulating hormones, inflammatory cytokines, and appetite changes in geriatric horses are needed to identify species-specific and conserved mechanisms of aging-related weight loss.

While the overall pain score did not differ between DAC and DCC horses, horses that died naturally exhibited significantly higher levels of depression and withdrawal compared to all other groups (DCC, NC, Y) reflected by the score’s subitems ‘position in their enclosure’ and ‘demeanour’. In elderly humans, depression is strongly associated with an increased risk of short- and mid-term mortality, with risk levels comparable to or even exceeding those of major health conditions such as myocardial infarction or diabetes [72–77]. Horses can exhibit behavioural and postural profiles that share striking similarities with depressive states in humans. These include indifference to environmental stimuli and human interaction, reduced selective attention, lower plasma cortisol concentrations, diminished sucrose intake (an indicator of anhedonia), reduced head and ear movements, and a fixed gaze [78–82]. However, associations between depressive symptoms and mortality risk in horses have not been previously explored or reported.

This study, like any longitudinal research conducted under field conditions, has limitations that should be considered when interpreting the findings. The control group of healthy horses was relatively small (*n* = 6), reflecting the specific population of the animal sanctuary, which by nature houses primarily animals with chronic health conditions. Despite the limited control sample size, the statistically significant differences observed between groups indicate that the sample was sufficient to detect meaningful effects. Nonetheless, future studies with larger control groups would be valuable to confirm and expand upon these findings. The sanctuary-based setting also introduces an inherent selection bias, as this population may not be representative of horses kept in different husbandry systems, such as privately owned pleasure horses, working horses, or those in performance contexts. Thus, while the sanctuary provides a unique opportunity for long-term, detailed observation of aging equines, further research is needed to assess the generalizability of these results across diverse equine populations and husbandry environments. In addition, while adjustments were made for major known confounders such as age and sex, residual confounding or multicollinearity cannot be entirely excluded and should be considered when interpreting the findings.

Euthanasia is intended to serve as a humane method to end an animal’s suffering by providing a peaceful and painless death, yet the decision to euthanise is often fraught with ethical and emotional dilemmas for animal owners and veterinarians alike [83–97]. These end-of-life decisions are rarely based solely on the animal’s quality of life (QoL), best interests, or medical and ethical considerations. Instead, they are frequently shaped by emotional, economic, social, and legal factors, which add layers of complexity, conflicting responsibilities and conflicts of interest to the decision-making process [83–97]. In this study, where the horses were owned by a sanctuary, end-of-life decisions could be made exclusively based on the animal’s best interests, highlighting the challenge of defining ‘best interest’ in the context of equine euthanasia.

Animals cannot weigh the future benefits of life-extending treatments against current suffering, nor can they autonomously request euthanasia [83–97]. Consequently, the ethical responsibility of making these decisions rests on caregivers and veterinarians. These decisions are guided by observable signs of quality of life, medical diagnoses, available treatment options, and prognoses, but they require complex and subjective predictions about the animal’s experiences and responses to interventions [83–97]. Continued life can only be justified if the resultant life is deemed worth living—a determination that hinges on a nuanced assessment of the animal’s QoL. Given that QoL is inherently subjective and deeply tied to an individual’s unique perception of their circumstances, evaluating whether another being’s life remains worth living is fundamentally constrained by the biases and limitations of external assessment [83–97]. The significant variability in an individual animal’s capacity to cope with seemingly similar health conditions and functional limitations further complicates these assessments.

Horses in this study that were euthanized due to inexorable decline in function or intractable pain irresponsive to treatment had similar indicators for most health parameters and time budgets than horses that died naturally, but had significantly worse lameness in trot and a greater deterioration in health status, retrospectively supporting the euthanasia decision. Delayed euthanasia, particularly in horses, is a well-recognized welfare issue, often resulting in prolonged suffering [90, 93]. Owners’ challenges in accurately assessing pain or declining QoL in their animals contribute to such delays. The body condition and musculoskeletal pain scores employed in this study may aid in the early identification of horses requiring close monitoring to maintain QoL. However, there is a critical need for validated QoL assessment tools and end-of-life decision-making frameworks tailored to equines. Such models could help identify horses approaching end-of-life, establish treatment goals, and define specific endpoints signalling when euthanasia is warranted and thus support timely, well-informed decisions to promote animal welfare at the end of life [90].

## Data Availability

The datasets generated and analysed during the current study are included in this published article (and its Supplementary Information files) or available from the corresponding author on reasonable request.
